# Supplementary material for: Differential Colonization and Mucus Ultrastructure Visualization in Bovine Ileal and Rectal Organoid-Derived Monolayers Exposed to Enterohemorrhagic Escherichia coli
Source: Int J Mol Sci. 2024 Apr 30;25(9):4914. doi: 10.3390/ijms25094914 (PMC11084217; doi:10.3390/ijms25094914)
Supplement: Supplementary file 1 [file ijms-25-04914-s001.zip › ijms-2971396-supplementary.pdf]

Differential Colonization and Mucus Ultrastructure Visualization in Bovine Ileal and Rectal Organoid-Derived Monolayers Exposed to Enterohemorrhagic *Escherichia coli*

Minae Kawasaki and Yoko M. Ambrosini *

Department of Veterinary Clinical Sciences, College of Veterinary Medicine, Washington State University, Pullman, WA 99164, USA; minae.kawasaki@wsu.edu

* Correspondence: yoko.ambrosini@wsu.edu

Supplementary Information

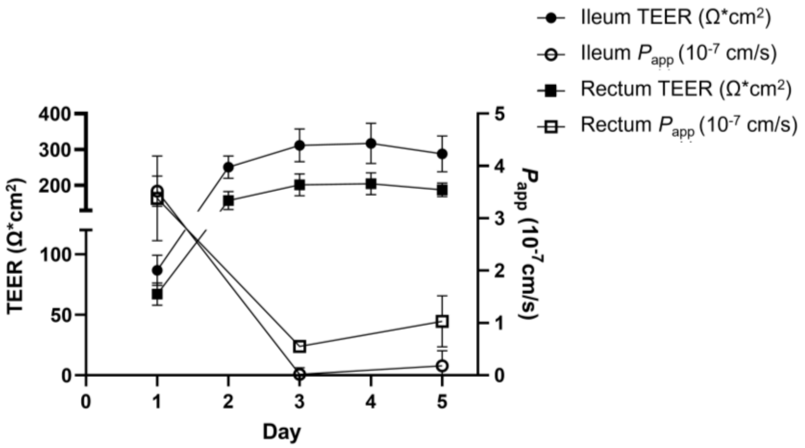

Figure S1: Development of stable bovine ileal and rectal organoid-derived monolayers prior to EHEC infection. Daily transepithelial electrical resistance (TEER) measurements and permeability (P_{app}) assay at Day 1, 3, and 5 using 4 kDa FITC-dextran were utilized to confirm establishment of functional epithelial barrier integrity. The results are presented as mean ± standard error of the mean (sem) from at least two technical and three biological replicates.

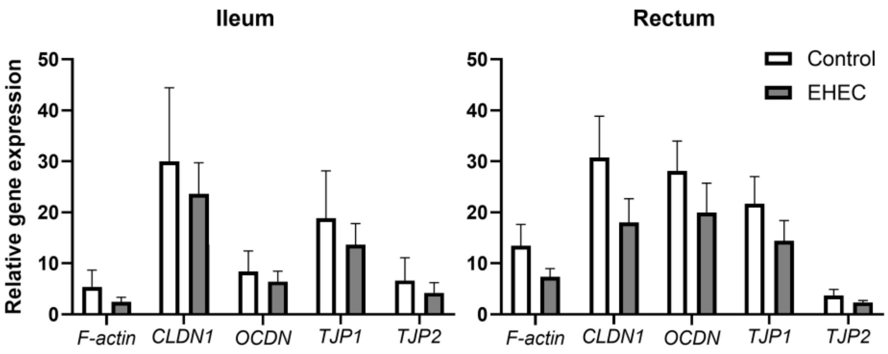

Figure S2. Impact of EHEC infection on expression of actin filament and junctional protein genes. RT-qPCR of control and EHEC-infected monolayers revealed no difference in expression levels of *F-actin*, *CLDN1*, *OCDN*, *TJP1* and *TJP2* genes between each other in both ileal and rectal monolayers at 4 h post infection. Relative gene expression levels were calculated using *GAPDH*, *RPL0* and *ACTB* as the internal control. Two technical replicates from three biological replicates were evaluated. Data are expressed as the mean ± sem.

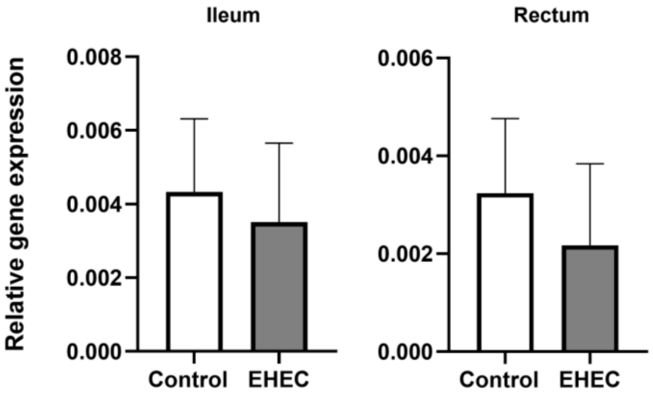

Figure S3. Relative gene expression levels of *MUC2* in control and EHEC-infected monolayers. RT-qPCR analysis revealed no differences between two groups in both ileal and rectal monolayers at 4 h post infection. Relative gene expression levels were calculated using *GAPDH*, *RPL0* and *ACTB* as the internal control. Two technical replicates from three biological replicates were evaluated. Data are expressed as the mean ± sem.

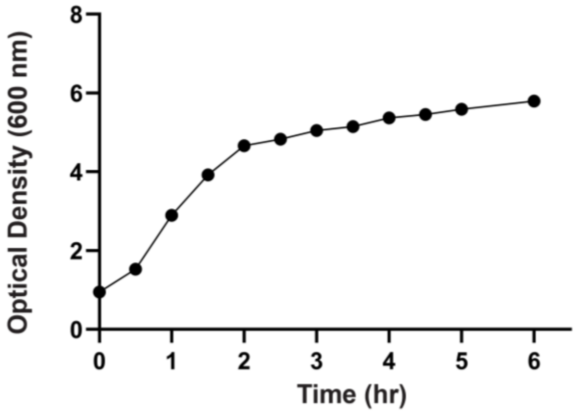

Figure S4. Growth profile of EHEC strain used in this study. The bacteria were grown in LB broth at 37 °C with shaking at 200 rpm. An overnight culture was diluted 1:10 into fresh LB broth at time 0.

Table S1. Culture media compositions for growth and maintenance of bovine ileal and rectal organoids and organoid-derived monolayers.

Reagent	Supplier	Final Concentration	Organoid Culture Medium	Ileal Monolayer Culture Medium	Rectal Monolayer Culture Medium
Advanced DMEM/F12	Gibco	NA	+	+	+
Noggin Conditioned Medium	N/A	10% (v/v)	+	+	+
R-Spondin Conditioned Medium	N/A	20% (v/v)	+	+	+
Recombinant Murine Wnt-3a	PeproTech	100 ng/mL	+	+	+
A-83-01	Sigma-Aldrich	500 nM	+	+	+
B27	Gibco	1x	+	+	+
Murine EGF	R&D Systems	50 ng/mL	+	+	+
Gastrin	Sigma-Aldrich	10 nM	+	+	+
N2	R&D Systems	1x	+	+	+
Nicotinamide	Sigma-Aldrich	10 mM	+	+	+
N-Acetyl-L-cysteine	MP Biomedicals	1 mM	+	+	+
SB202190	Sigma-Aldrich	10 µM	+	+	+
Primocin	InvivoGen	100 µg/mL	+	-	-
Penicillin/Streptomycin	Gibco	1x	+	-	-
GlutaMAX-I	Gibco	2 mM	+	+	+
HEPES	Gibco	10 mM	+	+	+
CHIR99021	Sigma-Aldrich	100 nM	+[1]	-	+
Y-27632	StemCellTechnologies	10 µM	+[1]	+	+
LY2157299	Sigma-Aldrich	500 nM	-	+	+
Fetal Bovine Serum	Gibco	20% (v/v)	-	+	+

[1] The reagent is withdrawn from the medium after the first 2-4 days of culture.
